# AAV-Containing Exosomes as a Novel Vector for Improved Gene Delivery to Lung Cancer Cells

**DOI:** 10.3389/fcell.2021.707607

**Published:** 2021-08-13

**Authors:** Bin Liu, Zhiqing Li, Shi Huang, Biying Yan, Shan He, Fengyuan Chen, Yaxuan Liang

**Affiliations:** ^1^Center for Biological Science and Technology, Advanced Institute of Natural Sciences, Beijing Normal University at Zhuhai, Zhuhai, China; ^2^Department of Cellular and Molecular Biology, Beijing Chest Hospital, Capital Medical University/Beijing Tuberculosis and Thoracic Tumor Research Institute, Beijing, China; ^3^Department of Burns, Nanfang Hospital, Southern Medical University, Guangzhou, China; ^4^Anhui University of Chinese Medicine, Hefei, China; ^5^Department of Pathology, School of Integrated Chinese and Western Medicine, Anhui University of Chinese Medicine, Hefei, China

**Keywords:** AAV-exosome, AAV, lung cancer, neutralizing antibody, gene therapy, exosome, extracellular vesicles

## Abstract

Lung carcinoma is the most common type of cancer and the leading cause of cancer-related death worldwide. Among the numerous therapeutic strategies for the treatment of lung cancer, adeno-associated virus (AAV)-mediated gene transfer has been demonstrated to have the potential to effectively suppress tumor growth or reverse the progression of the disease in a number of preclinical studies. AAV vector has a safety profile; however, the relatively low delivery efficacy to particular subtypes of lung carcinoma has limited its prospective clinical translation. Exosomes are nanosized extracellular vesicles secreted from nearly all known cell types. Exosomes have a membrane-enclosed structure carrying a range of cargo molecules for efficient intercellular transfer of functional entities, thus are considered as a superior vector for drug delivery. In the present study, we developed a novel strategy to produce and purify AAV-containing exosomes (AAVExo) from AAV-packaging HEK 293T cells. The cellular uptake capacity of exosomes assisted and enhanced AAV entry into cells and protected AAV from antibody neutralization, which was a serious challenge for AAV *in vivo* application. We tested a list of lung cancer cell lines representing non-small-cell lung cancer and small-cell lung cancer and found that AAVExo apparently improved the gene transfer efficiency compared to conventional AAV vector. Our *in vitro* results were supported *in vivo* in a lung cancer xenograft rodent model. Additionally, we evaluated the gene delivery efficiency in the presence of neutralizing antibody on lung cancer cells. The results demonstrated that AAVExo-mediated gene transfer was not impacted, while the AAV vectors were significantly blocked by the neutralizing antibody. Taken together, we established an efficient methodology for AAVExo purification, and the purified AAVExo largely enhanced gene delivery to lung cancer cells with remarkable resistance to antibody neutralization.

## Introduction

Lung carcinoma is the most common type of cancer and remains in the top rank of cancer-related mortality (Bray et al., 2018). Based on the cell size and appearance in histopathology, lung cancer is commonly categorized into two types including the non-small-cell lung cancer (NSCLC) and the small-cell lung cancer (SCLC), with the former accounts for more than 80% of lung cancers. Although chemotherapy and radiotherapy remain the standard treatment, they have serious side effects (Singh and Dhindsa, 2007). Gene therapy is defined as a therapeutic strategy introducing genetic materials into target cells. Up to date, the majority of clinical trials of gene therapy have targeted tumor tissues with delivered nucleotides expressed antiangiogenic factor, tumor suppressors, or immune stimulators for cancer treatment (Santiago-Ortiz and Schaffer, 2016). However, the selection of an ideal vector for gene delivery is still a major challenge. Adeno-associated virus (AAV) is a promising gene delivery vector for its safety, low toxicity, and multiple serotypes with preferred tropism to distinct tissue and cell types. Chen et al. (2017) observed suppression of NSCLC *in vivo* through delivery of Ang-(1–7) via AAV8, while another group suggested an AAV5-mediated strategy targeting mice bearing a xenograft A549 cancer (Wu et al., 2007). However, it was difficult to develop a best recombinant AAV for diverse lung cancer types, and the reported highest transduction efficiency was achieved between 30 and 50% at multiplicity of infection (MOI) of 100 (Chen et al., 2013), which was relatively low for an efficient clinical translation. The other major challenge that limits the efficacy of AAV-mediated gene therapy is the presence of serum-neutralizing antibody (Nab) that binds to AAV and blocks AAV infection (Nonnenmacher and Weber, 2012; Louis Jeune et al., 2013; Chaanine et al., 2014; Greenberg et al., 2015; Rapti et al., 2015). In fact, more than 90% of the human population is naturally infected with AAV, and about half has neutralizing antibody (Nab) against the virus (Louis Jeune et al., 2013). Nab binds to AAV capsid epitopes and inhibits their interaction with target cells (Nonnenmacher and Weber, 2011, 2012; Nonnenmacher et al., 2015), thereby reducing AAV transduction efficiency.

Exosomes are nanosized extracellular vesicles secreted from almost all cell types. Cargos carried within exosomes include specific proteins and RNAs that are transferred to recipient cells in the vicinity or at a distance (Liang and Sahoo, 2015). Recent studies indicate exosomes as a natural carrier for virus including hepatitis A and hepatitis C virus (Feng et al., 2013; Ramakrishnaiah et al., 2013). Interestingly, AAVs are also naturally secreted via exosomes (Maguire et al., 2012; György et al., 2014). Exosomes with their efficient rate of uptake by multiple cell types can assist the delivery of AAVs to target cells, and the naturally enveloped arrangement would shield the AAV vector from the neutralizing antibody. Therefore, AAV-containing exosomes (AAVExo) could be superior agents for delivering genes to lung cancer cells.

In this study, we developed a method of AAVExo purification based on iodixanol density gradient ultracentrifugation. Our purification methodology was able to effectively isolate AAVExo with minimal free AAV contamination. We compared the transduction rate of AAVExo with conventional AAV in several NSCLC cell lines (A549; H1299; adenocarcinoma HCC827, H23, and H1975; large-cell carcinoma H460; and H661) and SCLC cell lines (H446). We found that AAVExo has significantly higher transduction efficiency across all these cell types compared to the conventional AAV. Remarkably, AAVExo has superior resistance to Nab as compared to free AAV for lung cancer cell gene delivery. Our data support the improvement of AAVExo-mediated gene transfer both *in vitro* and *in vivo* and present a positive clinical prospect for lung cancer treatment in the future.

## Materials and Methods

### Cell Culture

HEK 293T cells were obtained from the American Type Culture Collection (Manassas, VA, United States) and cultured in Dulbecco’s modified Eagle’s medium (Cat. No. C11995500BT, Gibco, Paisley, Scotland, UK) supplemented with 10% fetal bovine serum (Cat. No. F8318, Sigma-Merck, United States) and 1% streptomycin–penicillin (Cat. No. V900929, Sigma-Merck, Shanghai Warehouse, China) in a humidified atmosphere with 5% CO_2_ at 37°C. A549, H1299, HCC827, H23, H1975, H460, H661, and H446 cell lines were obtained from the American Type Culture Collection (Manassas, VA, United States) and cultured in Roswell Park Memorial Institute (RPMI) 1640 medium (Cat. No. SH30809.01, HyClone Laboratories, South Logan, UT, United States) with the same supplements as that of HEK 293T cell culture.

### AAV and AAVExo Production and Purification

AAVs were produced by double transfection of HEK 293T cells as described previously (Rapti et al., 2012). Briefly, cells were cultured in a T175 flask with culture medium. When 60–70% confluency was achieved, cell culture medium was replaced with transfection reagent, which was made by mixing 50 μg of the helper plasmid, 17 μg of the transgene plasmid, and 233 μl of polyethylenimine (1 mg/ml, linear, MW ∼25,000; Cat. No. MX2202; Maokang, Shanghai, China) in Dulbecco’s modified Eagle’s medium (DMEM) supplemented with 2% fetal bovine serum (FBS) and streptomycin–penicillin. The cells were collected 3 days later at 300 *g* for 10 min (with cell-free supernatant saved for AAVExo purification) and resuspended in 10 ml of lysis buffer (150 mmol/l sodium chloride, 50 mmol/l Tris–HCl, pH = 8.5), subjected to three freeze–thaw cycles and treated with 1,500 U of benzonase nuclease (Cat. No. MP1509-25KU; Maokang, China) in the presence of 1 mmol/l magnesium chloride for 1 h at 37°C. Cellular debris was removed by centrifugation for 10 min at 5,000 *g* (Avanti J-E with a JA-25.50 rotor, Beckman Coulter, Brea, CA, United States). The virus was purified by a four-step iodixanol gradient centrifugation [5.8 ml of 15%, 3.9 ml of 25%, 3.1 ml of 40%, and 3.1 ml of 60% iodixanol (Optiprep, Cat. No. D1556; Sigma-Aldrich), overlayed with 10 ml of cell lysate in lysis buffer] in a 70Ti rotor (Beckman Coulter, Brea, CA) at 68,000 rpm for 1 h using polycarbonate bottles (Cat. No. 355618; Beckman Coulter). The 40–60% interphase of the gradient was collected, and the buffer was exchanged using a Vivaspin20 column with 100,000 MWCO (Sartorius, Göttingen, Germany) in sterile phosphate-buffered saline (PBS).

AAVExo were purified from cell culture medium by a combination of ultracentrifugation and Optiprep density gradient (Optiprep, Cat. No. D1556; Sigma-Aldrich). Specifically, a cell-free supernatant was sequentially centrifuged at 2,000 *g* and 10,000 *g* to remove cell debris and large vesicles. The supernatant from 10,000 *g* was ultracentrifuged under 100,000 *g* to obtain the crude exosome pellet, which was resuspended in 5 ml PBS and loaded on top of a four-step iodixanol gradient (5 ml of 15%, 10 ml of 25%, 3 ml of 40%, and 2 ml of 60%) and centrifuged at 250,000 *g* for 3 h using polycarbonate bottles (Cat. No. 355618; Beckman Coulter) and a 70Ti rotor (Beckman Coulter, Brea, CA, United States). Two milliliters of fractions from the top to the bottom of the gradient was collected. Fraction 6 that contained AAVExo was diluted in PBS and centrifuged at 100,000 *g* for 1 h. AAVExo pellet was resuspended in PBS for *in vitro* and *in vivo* experiments.

The titers of AAV and AAVExo were determined by quantitative PCR (qPCR) using the SYBR qPCR premix (PerfectStart Green qPCR SuperMix, Cat. No. AQ601-01, TransGen Biotech, Beijing, China) with an Applied Biosystems QS6 real-time PCR system (Applied Biosystems, Carlsbad, CA, United States) with primers against the CMV sequence (forward: 5′-TCAATTACGGGGTCATTAGTTC-3′; reverse: 5′-ACTAAT ACGTAGATGTACTGCC-3′). To test the plasmid contamina-tion, we used the primers targeting the Ampicillin resistance (AmpR) region (forward: 5′- CTCACCAGTCACAGAAAAGC -3′; reverse: 5′- AATGCTTAATCAGTGAGGCACC -3′).

### Nanoparticle Tracking Analysis for Exosome Size and Concentration

The ZetaView^®^ PMX 110 (Particle Metrix, Meerbusch, Germany) NTA instrument was employed to evaluate the exosome and AAVExo used in this study. Polystyrene nanoparticle standard (102 nm; Cat. No. 3100A, Thermo Fisher Scientific Inc., Waltham, MA, United States) was used for instrument calibration prior to each day’s analyses. Purified exosome or AAVExo or raw condition medium was serially diluted in PBS to provide optimal initial ZetaView^®^ instrument readings (10^6^–10^9^ particles/ml) and then evaluated for consistency over three measurement cycles. A set of parameters for data acquisition was standardized throughout the experiments: temperature of 23°C, sensitivity of 85, video frame rate of 30 frames per second, and a capture shutter speed of 100. Postacquisition parameters for exosome/AAVExo analysis included minimum brightness of 25, maximum size of 200 pixels, and a minimum size of 5 pixels (with pixel size not correlating to an equivalent nanometer diameter value). The data for nanoparticle diameters and concentration (particles/ml) from the ZetaView^®^ were analyzed using a proprietary software package (ZetaView^®^ 8.02.28) and graphically displayed and further analyzed via Excel.

### Electron Microscopy

AAV-producing HEK 293T cells were washed with PBS and fixed on the flask with 1% glutaraldehyde for 20 min. Then, cells were gently scrapped off the flask and washed with PBS, followed by 1% osmium tetroxide for 40 min at room temperature. Cells were then embedded in EPON resin (Electron Microscopy Sciences, Hatfield, PA, United States). Thin and ultrathin sections were cut on an ultramicrotome (Leica HistoCore MULTICUT) and stained with uranyl acetate and lead citrate. Samples were observed using a JEOL JEM-2100 plus microscope operating at 40–50 kV.

### *In vitro* Transduction

HEK 293T or lung cancer cell lines were seeded on a 48-well plate and cultured in DMEM or RPMI 1,640, respectively, with 10% FBS and penicillin/streptomycin. Cells were ready for AAVExo or AAV infection when they reached ∼70% confluency. Dilutions of mice serum (neutralizing antibody positive) from 1/5 to 1/160 or equal volume of PBS were mixed with AAVExo or AAV for 30 min at 37°C and then added to the cell culture. Three days later, cells were ready for fluorescence microscopy imaging.

### Fluorescence Imaging and Quantification

The fluorescence of mCherry/EGFP expression and 4′,6-diamidino-2-phenylindole (DAPI) staining was imaged by Zeiss Axio Observer 7. Five fields of view with four from corners and one from the center were snapped from each well. All images were captured at the same exposure setting. The transduction efficiency was expressed by the normalized intensity, which was extracted from the original grayscale images using ImageJ. Normalized intensity was calculated by normalizing the corrected mCherry/EGFP intensity (the mean intensity from mCherry/EGFP channel subtracting the mean intensity of the background) normalized to the corrected DAPI intensity (the mean intensity from DAPI channel subtracting the mean intensity of the background). The background intensity was defined by averaging the pixels within a selected region without fluorescence. The normalized intensities were averaged from five fields for each well, and three independent biological replicates were performed.

### Animals and Lung Tumor Xenograft Model

All animal experiments were approved by the Ethics Committee of Anhui University of Chinese Medicine and was in compliance with the institutional and governmental regulations. Male C57BL/6 and NOD SCID mice of 4–6 weeks old were purchased from Cyagen Biosciences, Guangzhou, China. For Nab-positive serum collection, C57BL/6 mice were intravenously injected with AAV6-EGFP (1E9 g.c.). Blood was collected after 48 h, and serum was obtained by clotting at room temperature and centrifuging at 2,000 *g* for 10 min. For tumor cell injection, 5 × 10^6^ A549 cells were subcutaneously injected into both dorsal flanks or one ventral site (in the following trial for other mice) of NOD SCID mice with a 21-G needle. Tumor growth was checked every week, and mice were ready to randomize into three groups (*n* = 1 for a preliminary trial, and *n* = 4 for the following reproducing trial) after 4 weeks. Equal titer (5E9 g.c. in total) and volume of AAV6Exo-luciferase, AAV6-luciferase, or saline was directly injected into multiple (three to four) sites of the tumor tissue. One week later, luciferase gene transfer and expression was examined through IVIS^®^ Spectrum optical imaging system (Lumina III, PerkinElmer, Waltham, MA, United States). Mice were anesthetized and then injected intraperitoneally with D-luciferin resuspended in PBS (150 μg/g body weight; Sigma). Postinjection mice were imaged for luciferase expression using an IVIS100 charge-coupled device imaging system every 2 min until the signal reached a plateau. Data analysis for signal intensities and image comparisons were performed using Living Image^®^ software (Caliper Life Sciences, Waltham, MA, United States). To calculate total flux in photons per second for each animal, regions of interest were carefully drawn and quantified around tumor areas.

### Statistics

All data were presented as mean ± standard deviation. One-way ANOVA was used to evaluate statistical significance in the mouse experiment by GraphPad. A *p* value was considered to be significance when < 0.05.

## Results

### Strategies for Purification of AAVExo With Minimal Contamination of Free AAVs

HEK 293T cells, which are widely used to generate AAVs, are known to secrete exosomes (Ban et al., 2015; Kanada et al., 2015). To test whether they also secrete AAV-containing exosomes (Maguire et al., 2012), HEK 293T cells were transfected with standard plasmids reported previously (Kho et al., 2011) to produce double-stranded AAV-EGFP. Consistent with the literature report (Vandenberghe et al., 2010), we found that the extracellular secretion of AAV6 was significantly higher compared to most other serotypes (data not shown). Therefore, we chose to use AAV6 in our subsequent experiments.

**FIGURE 1:**
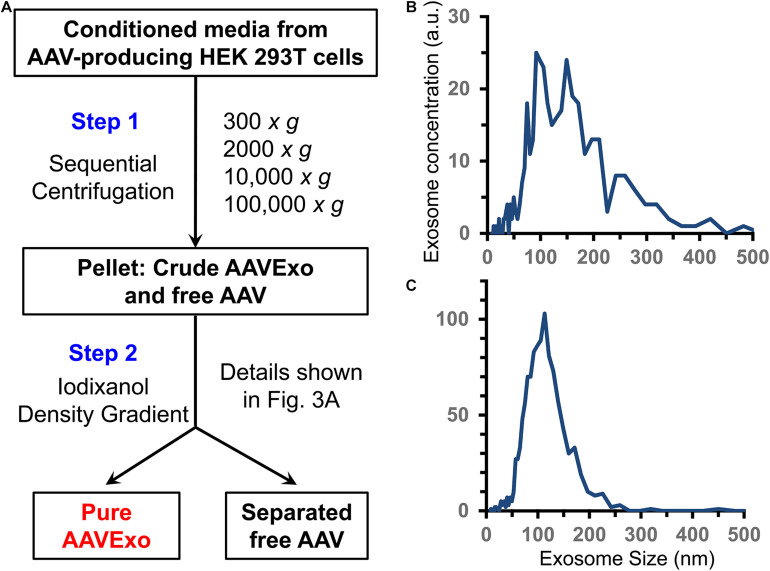
Exosomes are secreted and isolated from the conditioned medium of AAV-transfected HEK 293T cells. **(A)** Methodology flow chart of 2-step AAVExo purification. **(B)** Conditioned medium of AAV-producing HEK 293T cells analyzed by ZetaView. Five biological replicates were performed, and a representative was shown. **(C)** Purified AAVExo analyzed by ZetaView. Three biological replicates were performed, and a representative was shown here.

In addition to AAVExo, free AAV is released to culture medium as well (Vandenberghe et al., 2010). To isolate AAVExo without significant contamination with free AAV, we meticulously designed sequence of steps as shown in Figure 1A. We obtained crude AAVExo plus copelleted free AAV in Step 1 and subsequently collected pure AAVExo pellet by designing Step 2 with 15–60% iodixanol density gradient based on the flotation density of exosomes, AAVEXo, and free AAV. We compared the purity of AAVExo before and after the isolation using nanoparticle tracking analysis (ZetaView^®^ by Particle Metrix). The small and large particles and protein aggregates were observed in the raw conditioned medium population of AAVExo (∼100 nm) (Figure 1B), while after a two-step isolation clean population of AAVExo was obtained (Figure 1C).

**FIGURE 2:**
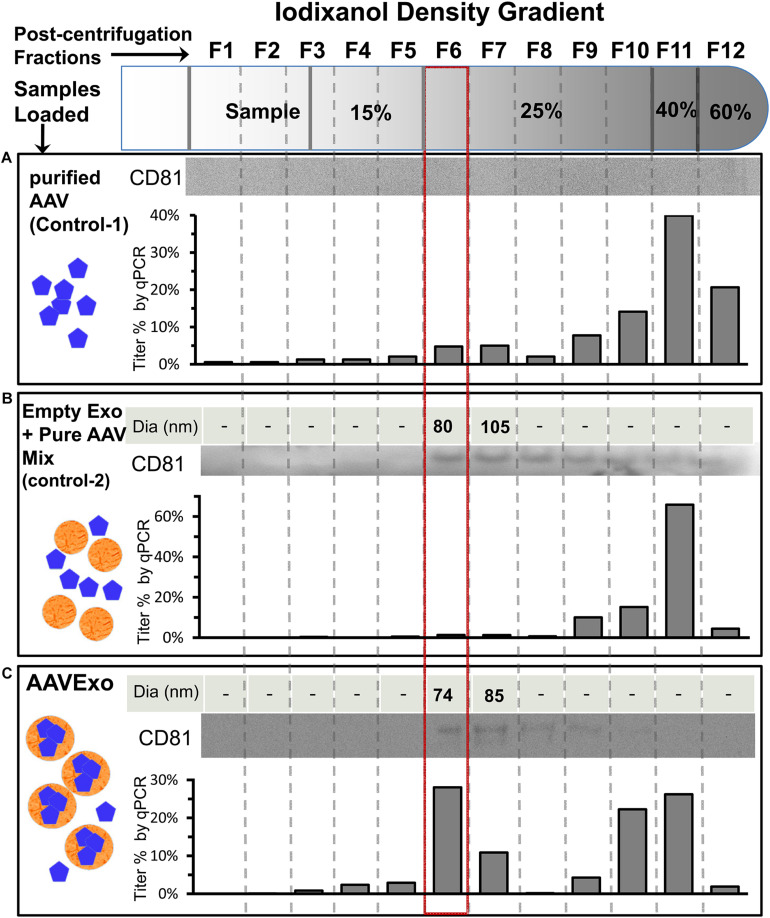
Strategies and characterization of AAVExo purification. Equal titer of pure AAV (**A**, control 1), a mixture of empty exo and pure AAV (**B**, control-2), and AAVExo **(C)** crude prep loaded on top of an Optiprep density gradient and ultracentrifuged at 250,000 *g* for 3 h. Separated fractions were collected from the top as indicated and analyzed for the presence of exosomes [(1) size, by ZetaView; (2) exosomes marker protein, CD81 by WB] and for the presence of AAV (by qPCR). The experiment was biologically reproduced for three times, and the representative results were shown.

Furthermore, we evaluated the purification efficiency of our density gradient-based isolation strategy by including sophisticated controls. Purified free AAVs were loaded as control 1 (Figure 2A) to demonstrate absence of free AAV in the AAVExo fraction. In addition, we included control 2, which was a mixture of empty wild-type exosomes and purified free AAVs (preincubated for 1 h, at 37°C before loading on the gradient) to demonstrate that free AAVs do not bind or stick to the surface of exosomes non-specifically (Figure 2B). Control 1, control 2, and AAVExo (Figure 2C) had equal amount of AAVs in genome copy (1E10 g.c.) number. Control 2 and AAVExo had equal amount of exosomes in vesicle number (characterized by ZetaView). After density gradient ultracentrifugation, we observed a white layer of exosomes floating at ∼20% in both the AAVExo and control 2 gradient but not in the control 1 gradient, which is in the location consistent with reported density of exosomes. Separate fractions with equal volume were collected from the top, and the layer with white band was precisely collected as fraction 6 (F6). Each fraction was analyzed for the presence of exosomes [(1) size by ZetaView and (2) exosomes marker protein CD81 by Western blot (WB)] and for the g.c. number of AAV by qPCR (Figures 2A–C). The ZetaView and WB data indicate that exosomes were primarily located in F6 (boxed in red) and F7. Additional exosome marker CD63 and flotillin-1 were confirmed within F6 and F7 (Supplementary Figure 1). Furthermore, a significant level of AAV genome was detected in F6 and F7 of AAVExo, while the free AAVs as shown in controls mainly presented downward in denser gradients. Since the primers that we used to detect the viral genome target the CMV sequence, it could be possible that the positive qPCR readings for AAVExo fractions came from the trace of original plasmid/PEI complex. To rule out this possibility, we designed a pair of primers targeting the ampicillin resistance (AmpR) region in the plasmid and performed qPCR for AAVExo fractions (Supplementary Figure 2). We did not collect positive signals from the AmpR amplification, confirming the specificity of the earlier qPCR detection. Taken together, our data suggested that free AAV was not residing in and around F6, and more importantly, free AAVs did not show tendency of stickiness to exosomes. These results demonstrated that that our purification process successfully separated the AAVExo from free AAVs secreted by HEK 293T cells.

**FIGURE 3:**
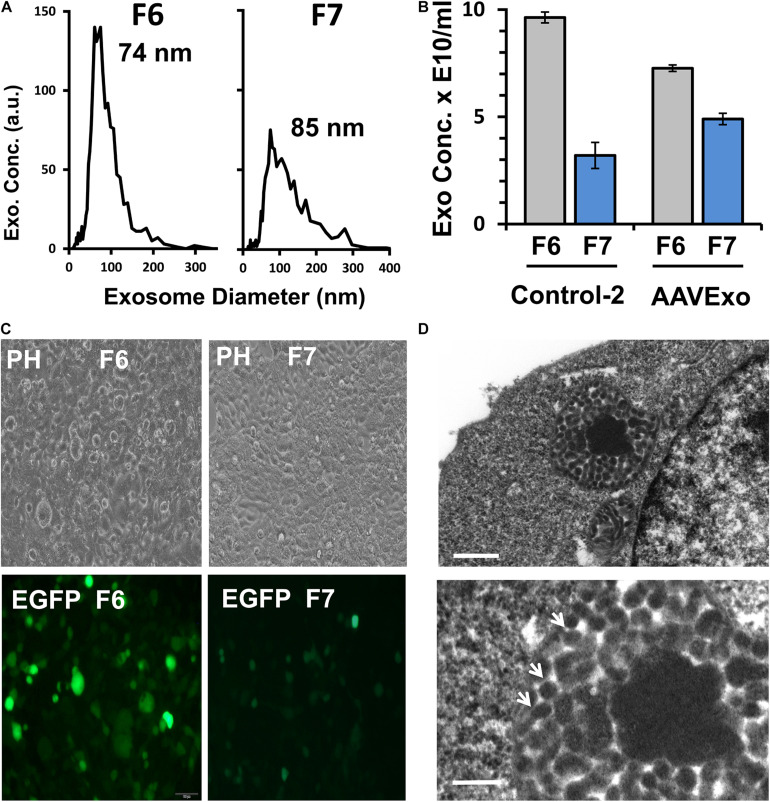
Fraction 6 has the major amount of clean AAVExo compared to fraction 7. **(A)** Exosome size distribution in F6 and F7 of AAVExo measured by ZetaView. Three replicates were conducted, and a representative was shown. **(B)** Exosome concentration of F6 and F7 in control 2 and AAVExo measured by ZetaView. The graph was plotted by the mean of three biological replicates with bars showing the standard deviations. **(C)** F6 but not F7 has effective transduction capacity *in vitro*. HEK 293T cells were treated with the same titer of AAV6Exo-EGFP F6 or F7 for 5 days. EGFP was detected by fluorescence microscopy. Three biological replicates were performed, and a representative was shown. **(D)** AAV-containing exosomes in multivesicular body. AAV-producing cells were imaged by TEM, and AAV particles were observed in exosomes prior to secretion. Lower panel represents the zoom-in area of upper panel. Scale bar: upper panel, 500 nm; lower panel, 200 nm.

As both F6 and F7 contained AAVExo, we analyzed them using ZetaView to determine the size and quantity of exosomes. AAVExo had comparable sizes in F6 and F7 (Figure 3A), while for quantity, F6 from both control 2 and AAVExo had 1.5–2.5-fold more exosomes than F7 (by particles/ml, Figure 3B). Moreover, the transduction efficiency of AAV6Exo-EGFP from F6 was significantly higher compared to that from F7 using HEK 293T cells (Figure 3C). In parallel, F9, F10, and F11 with pure AAV fractions were tested and showed moderate capacity of HEK cell infection (Supplementary Figure 3). Next, we confirmed AAVs packed in exosomes using transmission electron microscopy (TEM). AAV-producing HEK 293T cells were fixed and embedded in resin, of which the sections were imaged by TEM. Within the cytoplasm, we observed exosomes in the multivesicular body, a membrane-bound structure that fuses with plasma membrane and releases exosomes into extracellular environment. Notably, viral particles with viral DNA densely stained were found within exosomes, as indicated by arrows in Figure 3D. The morphology of purified AAVExo was further confirmed by TEM (Supplementary Figure 4). These data suggested that AAVExo purified from F6 was relatively pure without significant contamination of free AAVs. AAVExo from F6 had higher transduction efficiency compared to that from F7. Thus, we chose F6 as AAVExo for all our subsequent studies.

### AAVExo Has Higher Gene Delivery Efficiency Compared to AAV *in vitro*

**FIGURE 4:**
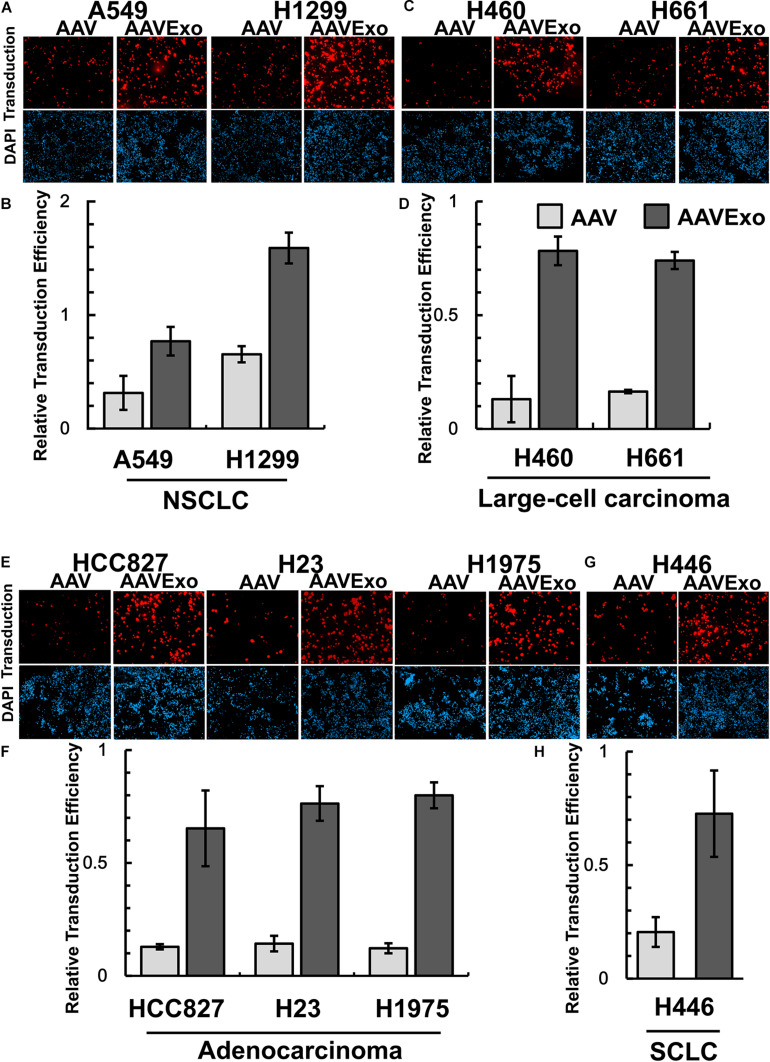
AAVExo has high capacity for gene delivery *in vitro*. A couple of selected lung cancer cell lines representing **(A,B)** common NSCLC, **(C,D)** large-cell carcinoma, **(E,F)** adenocarcinoma, or **(G,H)** SCLC were infected by equal titer (3E6 g.c. for a 48-well plate) of AAV6Exo-mCherry or pure AAV6-mCherry for 3 days. Cells were stained with DAPI before fluorescence microscopy imaging. The mCherry intensity was quantified and normalized to DAPI and plotted via Microsoft Excel. This experiment was reproduced biologically for three times, and the mean with standard deviation was graphed.

To compare the gene transfer efficiency of AAV and AAVExo, we produced double-stranded AAV6-mCherry and AAV6Exo-mCherry using the above protocol. A list of cell lines representing human lung cancer was selected from the NCI-60 panel as *in vitro* cell models to test AAVExo-mediated gene delivery. Specifically, the extensively used A549 and H1299 were included to represent common NSCLC; H460 and H661 were included as models for large-cell carcinoma in NSCLC; HCC827, H23, and H1975 were chosen to represent adenocarcinoma in NSCLC; and finally, H446 were incorporated into the experiment to represent SCLC. We covered a total of eight cell types for multiple cancer subtypes because the molecular mechanism of exosome uptake was not well known, particularly for the AAVExo uptake by lung cancer cells. Distinct cell types may display different surface protein profiles with varied preference for exosome docking, endocytosis, or membrane fusion. Therefore, we would like to verify the gene delivery efficiency of AAVExo within multiple types of lung cancer types. As stated above, AAV6Exo was selected due to the high yield of exosome-enveloped AAVs harvested from the cell culture supernatant. Equal tier of pure AAVs was set as a control. The intensity of mCherry expression was quantified and normalized to the DAPI intensity to represent the efficiency for gene delivery (Figure 4). We noted that the fluorescence expression was dependent on the total titer of administration (or multiplicity of infection, MOI) and the cell culturing time. Therefore, for all experiments, we fixed the MOI to 100 (equal to 3E6 g.c. virus per well) and the incubation time to 3 days. Remarkably, we found that AAVExo had a higher capacity for gene delivery across the common NSCLC cell lines (Figures 4A,B), large-cell carcinoma cell lines (Figures 4C,D), adenocarcinoma cell lines (Figures 4E,F), and a SCLC cell line (Figures 4G,H). The transfection enhancement was around two- to fourfold presenting in A549 and H1975. Collectively, the result demonstrated that AAVExo had significantly higher transduction efficiency than AAV to a variety of lung cancer cell types *in vitro*.

### AAVExo Is More Resistant to Antibody Neutralization Compared to AAV in Lung Cancer Cells *in vitro*

**FIGURE 5:**
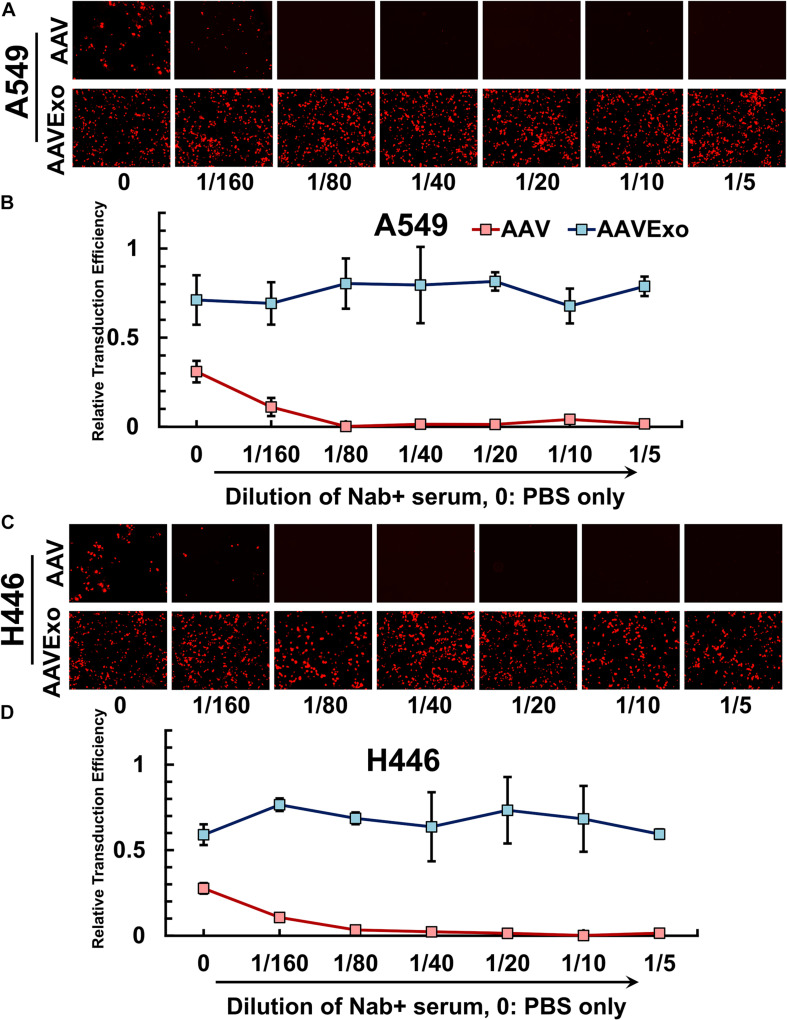
AAVExo has excellence resistance to neutralizing antibodies *in vitro*. Dilutions (1/5–1/160) of Nab-positive serum or PBS were incubated with AAV6Exo-mCherry or AAV6-mCherry for 30 min. The mixture with equal titer of genome copy was added to the culture of **(A,B)** A549 or **(C,D)** H446, and cells were ready for imaging 3 days later. Fluorescence intensity was quantified and normalized to DAPI for compiled plotting. The plot showed the mean with the standard deviation for three biological replicates.

Pre-existence of neutralizing antibody (Nab) against AAV is prevalent in the human serum. Nab binds to AAV, blocks its infection, and impairs AAV-mediated gene delivery. AAVExo has AAV enveloped inside the exosome compartment, therefore may have the potential to protect the AAV from antibody neutralization. To determine whether AAVExo is resistant to Nab neutralization, we generated Nab-positive serum by intravenously injecting AAV6-EGFP to C57BL/6 mice and collecting blood 48 h thereafter. The Nab-positive serum was shown to sufficiently suppress the AAV infection *in vitro* (data not shown). Equal titer of AAV6Exo-mCherry or AAV6-mCherry was preincubated with dilutions of Nab-positive serum or PBS control at 37°C for 30 min before applied on A549 or H446 cells. After 3 days, mCherry expression was examined by fluorescence microscopy, and the relative transfection efficiency was plotted from the quantified mCherry intensity that was normalized to the DAPI intensity (Figures 5A,B for A549 and Figures 5C,D for H446). As expected, we observed a significant reduction of mCherry expression for Nab serum-incubated AAV (∼0 at 1/40 dilution). However, there was no significant decrease in AAVExo-mCherry expression in the presence of Nab in selected lung cancer cell models. These data indicated that AAVExo, but not AAVs, can resist neutralization by the Nab.

### AAVExo Has Higher Capacity of Gene Delivery Efficiency to Lung Cancer Compared to AAV *in vivo*

**FIGURE 6:**
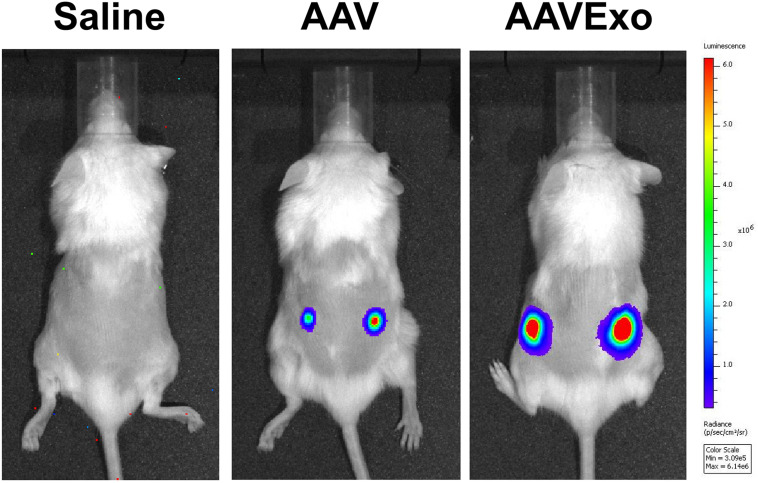
AAVExo has stronger gene-transfer efficacy *in vivo*. Male NOD SCID mice aged 4–6 weeks were subcutaneously implanted with A549 cancer cells. Four weeks later, equal titer (5E9 g.c.) of AAV6Exo-luciferase, AAV6-luciferase, or saline was directly injected into the tumor. One week later, bioluminescence was recorded by IVIS system.

Furthermore, we explored the AAVExo-mediated gene delivery in a rodent lung cancer xenograft model. In brief, A549 cells were subcutaneously implanted into the dorsal flank areas or ventral areas. The tumors were allowed to grow for 4 weeks followed by direct intratumoral injection of AAV6Exo-luciferase, AAV6-luciferase, or saline as a control. One week later, live mice were imaged for firefly luciferase expression through bioluminescent imaging. Mice enrolled in the first trial with tumors implanted on both sides of the dorsal flank areas are shown in Figure 6. Other mice administrated in a follow-up trial with ventral xenograft are shown in Supplementary Figure 5. Consistently, AAV6 administration exhibited a certain level of gene transfer and expression within tumor tissues as expected; however, AAV6Exo treatment demonstrated a significantly higher efficiency of gene delivery to the xenografts, which was consistent with the *in vitro* data presented above. We did not observe luciferase expression within other tissues, most likely due to the local injection performed, suggesting a rapid uptake of vectors by the tumor cell instead of dispersing in the systematic circulation. Taken together, these data demonstrated that AAVExo was a superior vector for enhanced gene delivery to the lung carcinoma than the conventional AAV vector.

## Discussion

AAV vectors have demonstrated their high safety profiles among over a hundred of early phase clinical trials worldwide (Kuzmin et al., 2021). Although a few AAV-mediated gene therapies have finally been approved (European Medicines Agency, 2012; Administration, U.S.F.a.D,2017,2019), effective gene treatment for patients with cancers is still not well developed. It comes to our attention that one of the reasons could be the low efficiency of tumor delivery for most AAV-based gene therapy, due to the weak tropism to tumor tissues, and the prevalence of AAV neutralizing antibody existed in human serum (Weber, 2021). Thus, it has become essential to improve the current gene transfer systems and reinforce delivery tools and methods for future success.

Recently, Maguire et al. (2012) and György et al. (2014) reported that AAVs were associated with extracellular vesicle (EV) and therefore acquired protection against inhibition by its neutralizing antibody. However, although it outperformed conventional AAV, EV-associated AAV was inhibited by higher concentration of Nab. Because EVs are a mixture of highly heterogeneous vesicles in their biogenesis, functions, and sizes, inadequate isolation could be a reason that lowers the effect of EV-AAV. Additional pitfall of compromised evasion from Nab might be the failure of excluding free AAV vectors from the final EV-AAV isolates. This is important because, while free AAVs count for the total titer of target genes, it will be eventually eliminated by Nab, thus impairing the overall effects of resistance to Nab. In our study, we devised a strategy to optimize the combination of ultracentrifugation and iodixanol density gradients and developed a novel method for isolating EV-AAVs with high purity and minimal contamination of free AAV vectors. More importantly, our study focused on exosomes, which are a population of EV with robust structure and smaller size ranging from 70 to 150 nm. To prove the effective purification of AAV–exosomes, we designed a meticulously controlled experiment, which included pure AAV vectors and premixture of wild-type exosomes and pure AAVs. The purpose of pure AAV control is to verify its distinct floating density against AAVExo, while the setup of premixture control is to address the concern that AAVs may potentially bind to the surface of exosomes non-specifically. With the examination of exosomal protein marker and viral genome, our data supported that nonspecific binding of AAV to exosome was minimal in our experiments. More importantly, only the AAVExo fraction with lowest possible contamination of free AAVs was successfully isolated. The development of effective purification method for AAVExo enabled the application of the new exosome-based vector to therapeutic treatments of lung cancers.

We characterized the purified AAVExo using several different *in vitro* systems to determine its performance in aspects of transduction and evasion from Nab. First, we thoroughly compared the transduction efficiency of AAVExo and conventional AAV vectors among a panel of lung cancer cell types. Remarkably, we observed highly enhanced efficiency of gene delivery and expression through AAVExo when compared to conventional AAVs. This result was robustly confirmed in multiple cultured lung cancer cell lines and in a xenograft mouse model. It is not fully understood why AAVExo could notably increase the transduction efficiency in the molecular level, and more work is needed to unveil the entry of AAVExo into recipient cells and its intracellular pathways in the future. Nevertheless, as a promising vector for cancer cell gene transfer, AAVExo has great potential to improve the strategies of gene transfer for the treatment of lung cancer.

Currently, one of the top challenges for AAV-based gene delivery is to overcome antibody neutralization, which is prevalent in the human body. There has been continuous effort to limit neutralization of AAV in multiple dimensions, including viral capsid engineering (Maheshri et al., 2006), pretreatment with anti-CD4 antibodies (Manning et al., 1998), or empty capsid decoys (Mingozzi et al., 2013). However, although promising, all of those approaches have limitations and drawbacks (Louis Jeune et al., 2013). AAVExo, as a novel vector, is thought to have virus protected by the exosome and is expected to evade Nab binding. Thus, we designed *in vitro* experiments to test the resistance of AAVExo to a dilution of increasing concentrations of Nab. We chose A549 and H446 as cell models for the transduction with AAVExo that had been preincubated with increasing concentration of Nab. We observed sustained AAVExo transduction without significant influence from increasing Nab, whereas infection of conventional AAVs dramatically reduced and eventually muted completely. These data demonstrated the superior profile of AAVExo as a novel gene-transfer vector with good resistance to Nab in an *in vitro* system, although further *in vivo* study using a Nab-positive rodent model may be essential to thoroughly characterize AAVExo transduction and resistance to Nab.

As an innovative approach, AAV-associated exosomes have been reported to efficiently deliver genes to the central nervous system (Hudry et al., 2016; Volak et al., 2018), immune cells (Breuer et al., 2020), retina (Wassmer et al., 2017), cochlea (György et al., 2017), and liver cells (Meliani et al., 2017). Our study has shown great potential that AAVExo may enhance the existing gene therapies for cancer treatment. In addition to the improvement in gene delivery efficacy and prevention of Nab neutralization that are presented in our study, exosome-based therapeutic platform has other substantial benefits. Published data from our and other laboratories demonstrated that exosomes could be engineered for selected cargo loading and the surface display of tumor-targeting entities (Ohno et al., 2013; Villarroya-Beltri et al., 2013; Liang et al., 2014; Kooijmans et al., 2016; Yim et al., 2016). On the other hand, AAVExo shields the natural tropism of AAV serotypes, and how serotypes affect AAVExo production and transduction warrants further exploration. On the other hand, although iodixanol density gradient provides the pure isolate, the potential scaling up in future manufactures should be considered. Despite the challenges, the future effort is worthwhile in designing an exosome-based gene therapy system that combines recombinant AAVs and vesicle surface engineering for lung-cancer-specific targeting.

## Data Availability Statement

The raw data supporting the conclusions of this article will be made available by the authors, without undue reservation.

## Ethics Statement

The animal study was reviewed and approved by Ethics Committee of Anhui University of Chinese Medicine.

## Author Contributions

YL conceived the study and the entire research plan. FC and SHu led and designed the animal experiment. BL conducted the *in vitro* and *in vivo* experiments. BY and SHe performed cell culture and AAV and AAVExo production and purification. YL and ZL wrote the manuscript and prepared the figures. All authors contributed to the article and approved the submitted version.

## Conflict of Interest

The authors declare that the research was conducted in the absence of any commercial or financial relationships that could be construed as a potential conflict of interest.

## Publisher’s Note

All claims expressed in this article are solely those of the authors and do not necessarily represent those of their affiliated organizations, or those of the publisher, the editors and the reviewers. Any product that may be evaluated in this article, or claim that may be made by its manufacturer, is not guaranteed or endorsed by the publisher.
