# Supplementary material for: AAV-Containing Exosomes as a Novel Vector for Improved Gene Delivery to Lung Cancer Cells
Source: Front Cell Dev Biol. 2021 Aug 13;9:707607. doi: 10.3389/fcell.2021.707607 (PMC8414974; doi:10.3389/fcell.2021.707607)
Supplement: Supplementary file 1 [file Data_Sheet_1.docx]

Supplementary Figures

AAV-Containing Exosomes as a Novel Vector for Improved Gene Delivery to Lung Cancer Cells

Bin Liu^1,2^†, Zhiqing Li^3^†, Shi Huang^4^†, Biying Yan^1^, Shan He^3^, Fengyuan Chen^5^*, Yaxuan Liang^1^*

1 Center for Biological Science and Technology, Advanced Institute of Natural Sciences, Beijing Normal University at Zhuhai 519087, China.

2 Department of Cellular and Molecular Biology, Beijing Chest Hospital, Capital Medical University/Beijing Tuberculosis and Thoracic Tumor Research Institute, Beijing, 101149, China;

3 Department of Burns, Nanfang Hospital, Southern Medical University, Guangzhou, 510515, China

4 Anhui University of Chinese Medicine, Hefei, 230031, China.

5 Department of pathology, School of Integrated Chinese and Western Medicine, Anhui University of Chinese Medicine, Hefei, 230031, China.


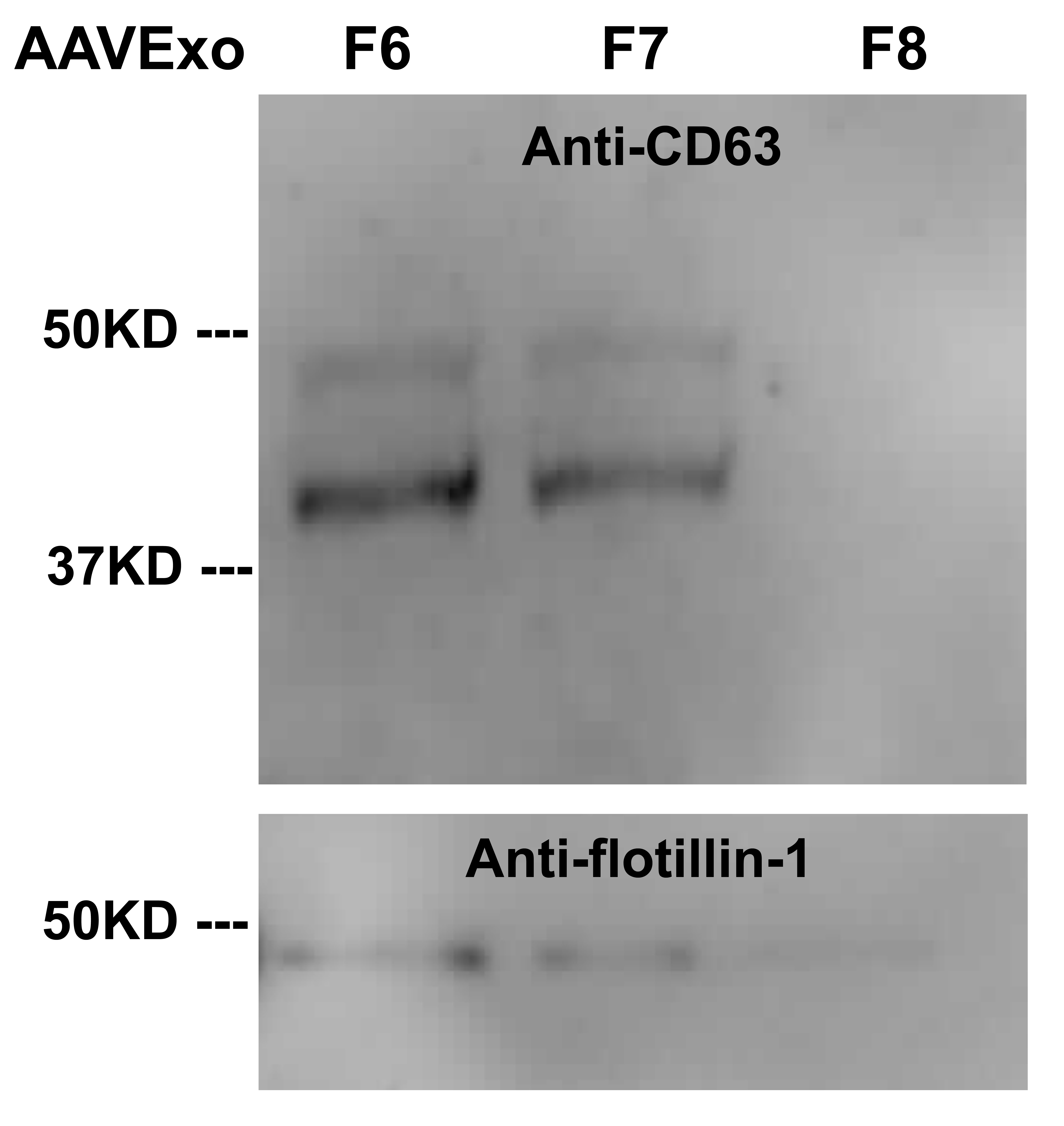


**Figure S1** Additional exosome markers CD63 and flotillin-1 was confirmed in F6 and F7 of AAVExo gradient.


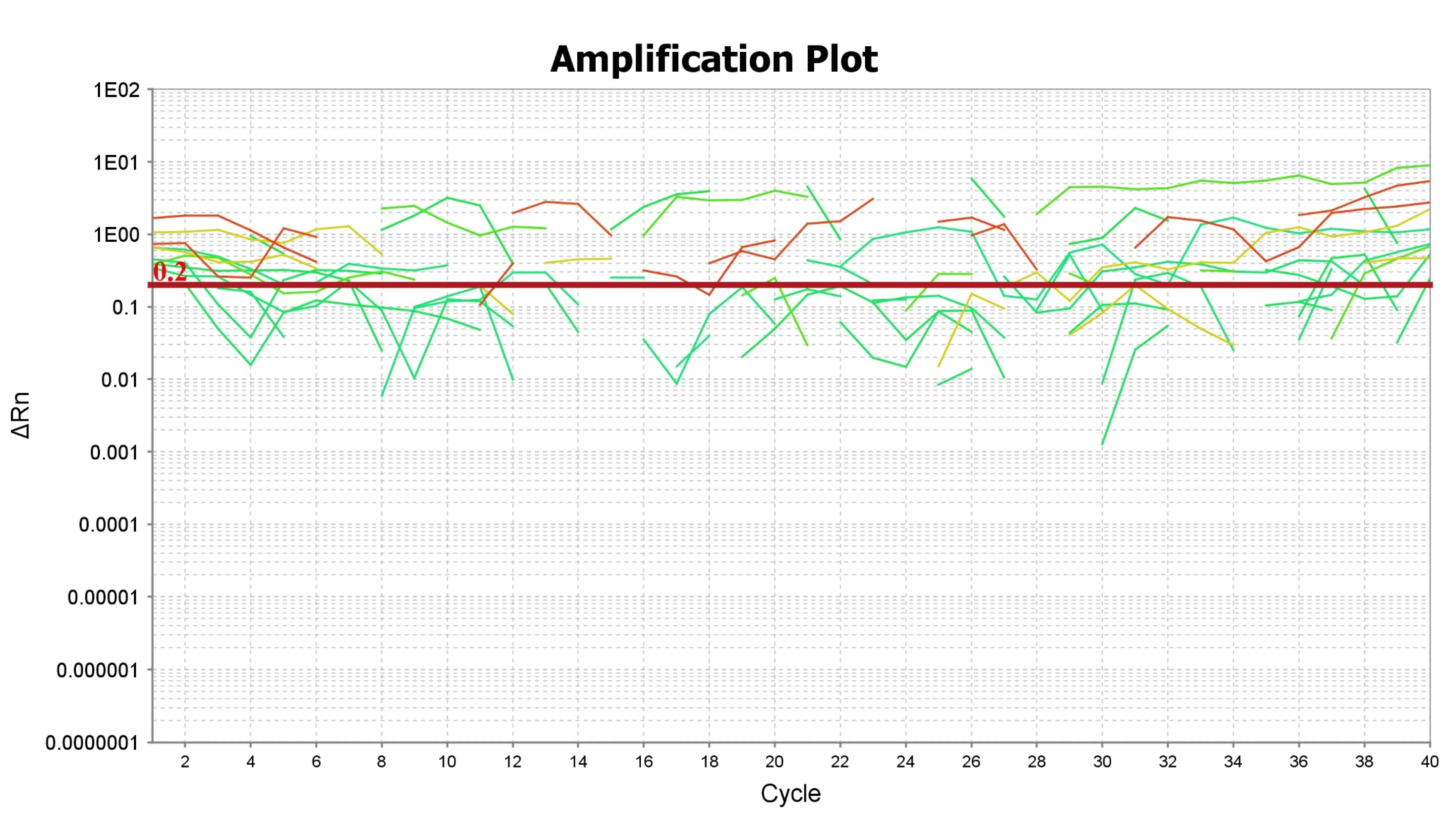


**Figure S2** Potential contamination from transfected plasmid was examined through qPCR targeting the AmpR region. A representative showed that no amplification was detected from the F5 (red), F6 (green), F7 (yellow), and F8 (blue).


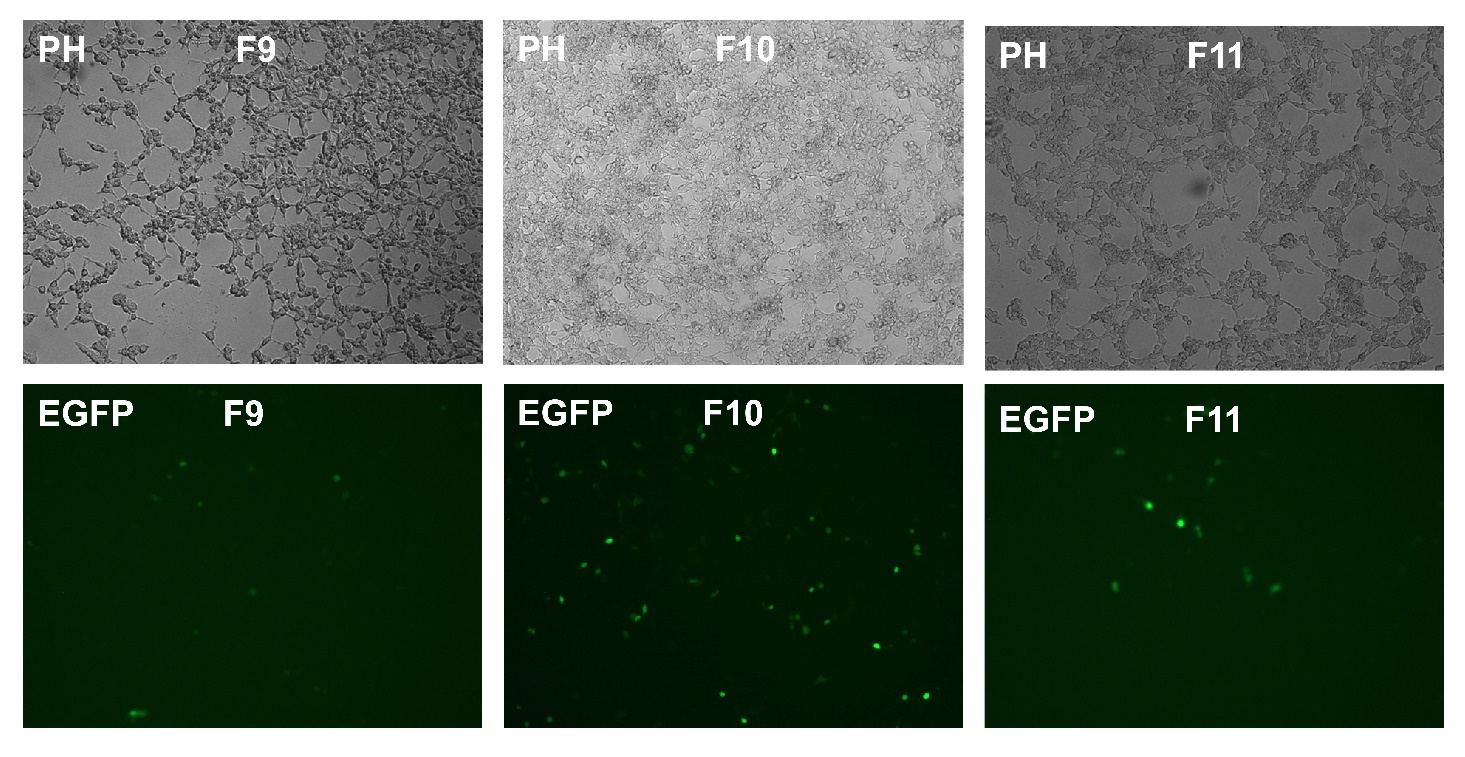


**Figure S3** Transduction of AAVs from F9, F10 and F11 were tested on HEK 293T cells. Consistent to Figure 2C, F10 and F11 showed certain level of gene expression.


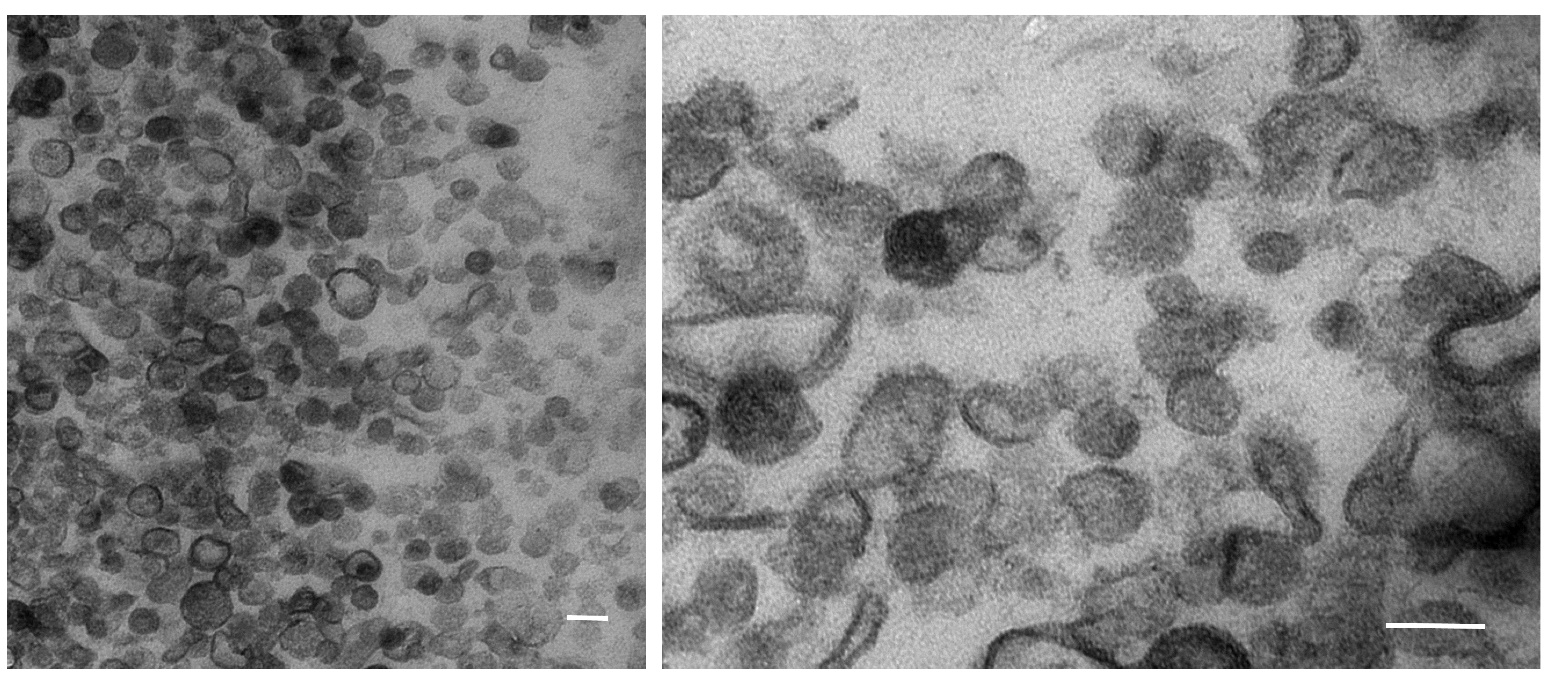


**Figure S4** Morphology of purified AAVExo by TEM. Scale bar, 200 nm.


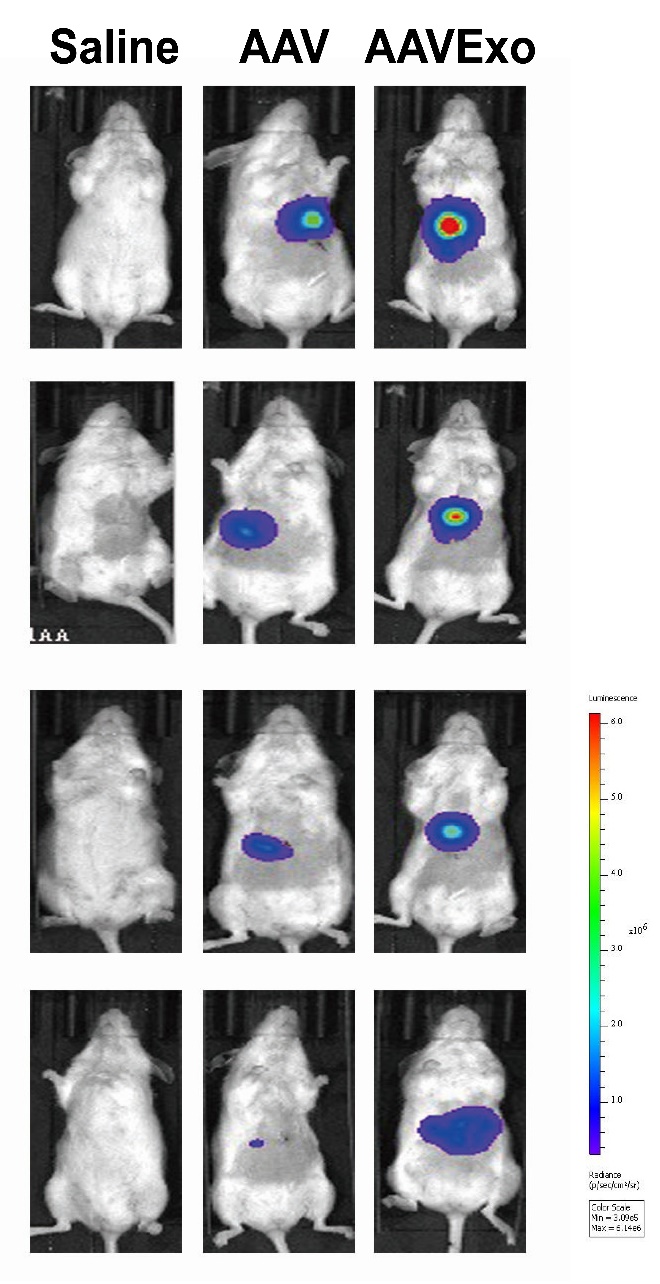


**Figure S5**.

Xenograft mice enrolled in a follow-up trial demonstrated the improved gene transfer efficacy for AAVExo. Male NOD SCID mice were subcutaneously implanted with A549 cells on the ventral side for 4 weeks. Equal titer (5E9 g.c.) of AAV6Exo-luciferase, AAV6-luciferase, or saline was directly injected into the tumor. One week later bioluminescence was captured by IVIS system.
